# Exploring retro-cue effects on visual working memory: insights from double-cue paradigm

**DOI:** 10.3389/fnins.2023.1338075

**Published:** 2024-01-11

**Authors:** Ruiqiao Guo, Junbo Wang, Kai Fu, Qiang Liu

**Affiliations:** ^1^Institute of Brain and Psychological Science, Sichuan Normal University, Chengdu, China; ^2^Research Center of Brain and Cognitive Neuroscience, Liaoning Normal University, Dalian, China

**Keywords:** double-cue, retro-cue benefit, CDA, VWM, passive state, active state

## Abstract

In the realm of visual working memory research, the retro-cue paradigm helps us study retro-cue effects such as retro-cue benefit (RCB) and retro-cue cost (RCC). RCB reflects better performance with cued items, while RCC indicates poorer performance with uncued items. Despite consistent evidence for RCB, it’s still uncertain whether it remains when previously uncued items are cued afterward. Additionally, research findings have been inconsistent. This study combines prior experiments by controlling the proportion of cue types and the number of memory items. Besides, using a CDA index to assess the status of items after the cue appeared. Results showed better performance under the double-cue condition (involving two cues pointing inconsistently with only the second cue being valid) compared to the neutral-cue condition, and better performance under the single-cue condition compared to double-cue. EEG data revealed that after the appearance of the second cue in the double-cue condition, there was a significant increase in CDA wave amplitude compared to the single-cue condition. Behavior results suggests that RCB occurs under double-cue but to a lesser extent than the single-cue. And EEG outcomes indicates that individuals did not remove the uncued item from their visual working memory after the first cue. Instead, they kept it in a passive state and then shifted it to an active state after the appearance of the second cue.

## Introduction

Visual working memory (VWM), a system designed to retain and manipulate visual information, presents an intriguing platform for exploring the interplay between limited capacity and flexible selection mechanisms under internal attention. The retro-cue paradigm has emerged as a valuable tool for investigating the impact of internal attention on VWM. In this paradigm, a retro-cue is introduced after the disappearance of visual information earmarked for memorization, directing attention to the item targeted for recall during subsequent testing.

Research within this framework has led to the identification of the retro-cue effect (RCE; Griffin and Nobre, 2003), which encapsulates both retro-cue benefit (RCB) and retro-cue cost (RCC). RCB denotes the observable advantage in behavioral responses when a valid retro-cue is presented, signifying an improvement in subjects’ performance subsequent to directing their internal attention towards items indicated by the cue within VWM. This phenomenon underscores the intricate relationship between VWM and internal attention. Besides, RCB also highlights the dynamic nature of the information within VWM, which is susceptibility to variations in internal attentional focus and hence, the accuracy of the item is also variable. Conversely, RCC elucidates the decline in memory representations for uncued items following the presentation of an invalid cue. This decline manifests as an increase in reaction time and a decrease in accuracy compared to the no-cue condition (Griffin and Nobre, 2003), offering insights into the repercussions of diverting internal attention within visual working memory.

Research indicates that retro-cues consistently induce a retro-cue benefit (RCB) effect when employed to orient internal attention (Poch et al., 2017; Wolff et al., 2017). However, limited investigation has focused on whether previously uncued items, when subsequently targeted by internal attention (via a second cue in the double-cue paradigm, different from the first), yield RCB effects. Three studies have delved into this question. Li and Saiki (2014) observed an emergence of RCB when both cues in a double-cue were 33% valid, demonstrating improved performance under the double-cue compared to the neutral-cue, suggesting that items, not previously cued, performed better when recued compared to the no-cue trial. Increasing the validity of the second cue to 50% and decreasing the first cue’s validity to 17% still yielded consistent results. However, in this study, due to low cue validity, subjects tended to distrust first cue and retain other items in memory when the first cue appeared. In van Moorselaar’s et al. (2015) Experiment 3, with 100% cue validity and an invalid first cue when a second cue appeared, double-cue performance was comparable to the no-cue, suggesting no RCB effect. Conversely, another research reported that performance under double-cue was superior to no-cue, indicating the presence of an RCB effect under the double-cue condition (Rerko and Oberauer, 2013). These findings underscore the complex interplay of internal attention and retro-cue effects, shedding light on the nuanced mechanisms at work.

In studies where both cues had a validity of 100%, divergent outcomes could be attributed to two potential reasons. In the study by van Moorselaar et al. (2015), where only 1/6 of trials involved double-cue and 1/2 involved single-cue, subjects tended to prioritize the first cue, concentrating the majority of their attention on the corresponding item while allocating fewer resources to encode the remaining items. Consequently, when the second cue appeared, the prior inadequate encoding meant that even with full attention directed to an item under the second cue, it could not undergo thorough processing. Consequently, the precision of extracted items was relatively low under the double-cue condition, comparable to the performance under the no-cue condition, where resources were evenly distributed among items. In contrast, Rerko and Oberauer’s (2013) study featured three cues with equal proportions of trials upon the disappearance of the first cue. This equality in the likelihood of a second cue appearing or not diminished any tendency to disproportionately focus resources on the initially cued item. Consequently, resources were retained for the uncued item to be processed and encoded upon the subsequent appearance of the second cue. Consequently, their study revealed a retro-cue benefit (RCB) effect for items not cued by the first cue but subsequently cued by the second cue.

Moreover, an individual’s working memory span typically encompasses 3 to 4 items (Luck and Vogel, 1997; Vogel et al., 2001; Zhang and Luck, 2011; Lewis-Peacock et al., 2018; Schneegans et al., 2020). When the memory array exceeds this capacity, some items inevitably exceed the threshold of retention within working memory. Consequently, if a cue directs attention to an unmaintained item, not only does the individual struggle to process and encode it accurately via internal attention, but they also encounter challenges during the recall phase. As the initial number of stimuli increases, the likelihood of items surpassing working memory capacity rises, amplifying the potential for the cue to target an unretained item, and subsequently diminishing its efficacy. In the study by van Moorselaar et al. (2015), the memory arrays comprised eight items, while the Rerko and Oberauer (2013) study featured six items. As a result, a larger proportion of items in the van Moorselaar et al. (2015) experiment exceeded working memory capacity, subsequently reducing the probability of a cue aligning with an item retained in working memory. This contextual discrepancy may underpin the inability of van Moorselaar et al. (2015) to observe a double-cued RCB contrasted with the findings of Rerko and Oberauer (2013). To address this, our present study restricted the memory array to four items, maintaining an equitable distribution of trials across all three cue types within a block. We predicted that an RCB effect would emerge for double-cue in this case.

In our study, EEG recordings were employed to elucidate the status of items cued by different cue types and their evolution in storage state throughout the task. This approach not only afforded insight into the mechanisms underlying the emergence or absence of the retro-cue benefit (RCB) effect under double-cue, but also provided a peek into the internal workings of visual working memory (VWM). Our findings relate to the “activity-silent” model, which describes dual states of VWM content storage: an active state engaged in ongoing processing and possessing a priority status, allowing representations to be actively sustained with neural activity, and a passive state devoid of ongoing processing and priority status, maintaining memory representations primarily through weight-based synaptic changes. Within the context of the sequential-encoding retrieval task, array representations irrelevant to the ongoing task assume a passive state, while those pertinent to current encoding or forthcoming probes reside in an active state. This model sheds light on the dynamic nature of VWM operations and the allocation of resources based on task relevance.

The CDA components have been widely acknowledged for their efficacy in dynamically tracking the number of memory items in an online-state working memory (Vogel et al., 2005; Ikkai et al., 2010; Gao et al., 2011; Ye et al., 2014; Feldmann-Wüstefeld et al., 2018). Specifically, as the memory representation transitions from a passive state to an active state, the CDA amplitude demonstrates a concurrent increase corresponding to the number of memory items, while the reverse transition from an active state to a passive state results in a rapid decrease and eventual disappearance of the CDA amplitude. For instance, in a study involving consecutive presentations of two memory arrays, no discernible difference in CDA amplitude between the two arrays was noted, suggesting that the first array had shifted to the passive state before the presentation of the second array (Li et al., 2020). This study underscores the utility of CDA as an effective indicator of the number of active stores, with a larger amplitude corresponding to a greater number of stores. Consequently, in our investigation, we employed this metric to meticulously track shifts in the item storage state.

Based on the results of a small number and conflicting previous studies, the present paper controlled for possible influencing variables (cue type ratio and number of memorized items) to further explore whether the RCB effect still occurred when an uncued item was cued again and also to use EEG techniques to reveal the effects of different cue types on the storage status of the item. Since this paper used the CDA metric, which requires the use of a lateralized stimulus, compared with the previous experimental procedure, in the present study, a pre-cue appeared before the stimulus presentation, instructing subjects to pay attention to one side of the stimulus in the left and right visual fields. Specifically, the experimental procedure was as follows: an pre-cue was presented first, pointing to a memory array (four) that did not exceed the working memory capacity on one side, and then three cue types of retro-cues were randomly presented to indicate the upcoming location of the item, including single-cue, neutral-cue, and double-cue (the second cue was valid and the first one was invalid), all with the same presentation probability. Finally, subjects were required to judge whether the color item at the corresponding locations in the probe stimuli was identical to the memory array, and the behavioral data of the subjects were recorded throughout the experiment. In the neutral-cue condition, subjects did not know which item would be probed and would adopt the strategy of keeping all items in working memory. Due to the limit of resources, each item would receive relatively small resources, and the precision of detection would be comparatively low. In the cued condition, on the other hand, before the presentation of the first retro-cue, the same as the neutral-cue condition, subjects kept all items in working memory. But after the first cue disappeared, there was a 50 percent chance that the cued item would be probed, and the individual would process and encode it with at least half of the resources. If only one cue is presented, the cued item receives more cognitive resources compared to the neutral-cue, and we predicted, consistent with previous research, that it would outperform the neutral-cue in terms of accuracy and response time, with an RCB effect. If a second cue was present, the first cue was invalid, the second cue must be valid, and the individual would release cognitive resources from the first cue and, in combination with the resources used for the passive state, focus them all on the item indicated by the second cue. At this point, the cognitive resources gained under the double-cue are similarly higher than under the neutral-cue, and we predicted that the accuracy and response time to the second cued item would also be better than under the neutral-cue, with the same RCB effect occurring.

In addition to behavioral data, subjects’ EEG responses are recorded throughout the experiment. It has been shown that in a retro-cue paradigm, cued items are retained in the active state during the delay after cue onset, while others items are stored in the passive state (Lewis-Peacock et al., 2012; LaRocque et al., 2013; Rose et al., 2016). However, those researches were based on single cue conditions, as we know, there has not been any research using EEG to uncover the storage status of items under double cue conditions. That is, it is unclear that whether the storage state of uncued items changes after they are cued again; and if it does, how it changes. Therefore, we also use the CDA index to follow the item’s storage state under different cue types and explore whether and how the uncued item changes its state after cued again. We hypothesized that after the first cue appeared, the cued item would go into the active state and the remaining items would go into the passive state, at which point there should be a change in CDA amplitude. Since subjects did not know whether a second cue would appear, individuals kept the first cued item in the active state all the time. If there was only one cue, the CDA amplitude would suddenly increase when the first cue appeared, and then it would gradually decrease. If a second cue appeared after the 800-ms interval, the original cued item would move from the active state to the passive state, and the new cued item would enter the active state from the passive state. At this point, in the time period after the second cue appeared, the double-cue CDA amplitude was improved compared to the single-cue.

## Method

### Subject

Referred to previous study about CDA component, there was a large effect size on the manipulation of items number (Feldmann-Wüstefeld et al., 2018). Thus, we predicted a medium-high effect size (effect size = 0.65) for our experimental design. With a significance level of 0.05 and a statistical power of with 0.8, the suggested total sample size was approximately13 participants. All subjects were recruited voluntarily for this experiment, and a total of 18 subjects (6 males, 12 females, all right-handed) participated in this experiment, and all of them were confirmed to be free of color blindness and color retardation, with normal visual acuity and corrected visual acuity in the naked eye. The age of the subjects was between 18 and 26 years old, with a mean age of 22.51 ± 2.13 years old. Each subject was paid $30/h for completing the experiment. Three of the subjects had behavioral outcomes below the random level and were not counted in the overall results. Thus a total of 15 subjects’ data were analyzed.

### Experimental materials

E-Prime 2.0 professional software was used to prepare the experimental program for the retrospective cueing paradigm. The memory array consisted of 8 color blocks, and the positions of the color blocks were always constant. Drawing on Kuo et al. (2012), the exact location and size of the color blocks were calculated based on human cortical magnification. The positions were presented on two virtual concentric circles with viewing angles of 3.06° and 5.44°, and the sizes of the color blocks were 0.77° and 1.36°, respectively. The colors of the color blocks were randomly selected from eight high-resolution colors: red, yellow, blue, green, magenta, purple, orange, and lime green. The stimuli were presented on a 19-inch CRT monitor, with subjects at a distance of 70 cm from the monitor, and the experiment was completed individually in a quiet and noiseless experimental room.

### Design

**Figure 1:**
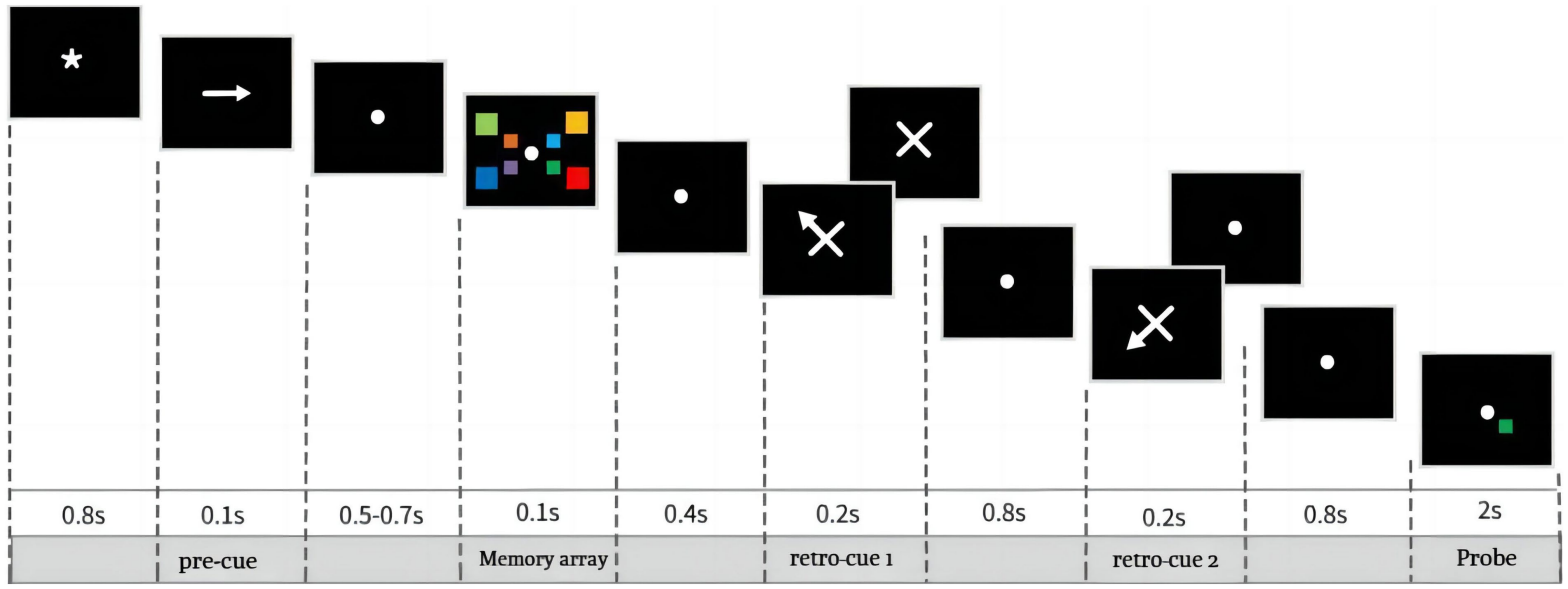
The procedure of experiment. The black background first displayed an 800 ms gaze point, succeeded by a leftward or rightward pointing arrow displayed for 100 ms, indicating which side of the field should be paid attention to. Subsequently, following a 500–700 ms blank interval, a memory array persisted for 100 ms, followed by a 400 ms delay and then 200 ms of retrospective cue, either neutral (1/3) or spatial (2/3) appearing randomly. After that, an 800 ms interval ensued. Subsequently, there was a second neutral (2/3) or spatial (1/3) retrospective cue appearing. Ultimately, participants were tasked with discerning whether the color block in the probe stimulus, corresponding to the location highlighted in the memory array, matched, indicated by pressing the “f” key for a match or the “j” key for a mismatch. The cue types including neutral-cue, single-cue, and double-cue, all of them have the same probabilities and, in the double-cue condition, the first cue is invalid and the second cue is 100% valid.

The experiment was divided into a practice experiment and a formal experiment. The practice experiment required subjects to complete 30 trials in which neutral-cues, single-cue, and double-cue appeared randomly, each accounting for one-third of the total trials. Feedback was presented at the end of each trial, prompting subjects to indicate whether the current trial was judged correctly. Once subjects understood the experimental procedure, they pressed the Q button to start the formal experiment, which did not include feedback. As shown in Figure 1, the black background first displayed an 800 ms gaze point, followed by an arrow pointing to the left or the right. The arrow was presented for 100 ms, instructing subjects which side of the gaze point the color block was task-relevant. The chance of the arrow pointing in the left or right direction was equal and randomized. After a 500–700 ms blank screen, a memory array appeared on the screen: four color blocks on each left and right side. However, subjects were only required to memorize the 4 color blocks on the side to which the arrow had previously pointed. The memory array was presented for 100 ms followed by a 400 ms interval, followed by 200 ms of cue, which could be either neutral or spatial cues, both appearing randomly. After the disappearance of the first cue is an 800 ms interval, there is a one-in-two chance that a second retrospective cue will appear, and the item pointed to by the second cue must not be the same as the item pointed to by the first cue, and the last cue that appears must be valid. If the trial contains only one cue, the cue must be valid; if the trial contains two cues, the second cue must be valid. The three conditions, neutral-cue, single-cue and double-cue, appeared equally and randomly within a set of trials, and subjects could not predict in advance how many cues would be included in that trial. Finally, subjects were asked to determine whether the color block at the corresponding location in the probe stimulus was the same as the memory array, pressing the f key for the same and the j key for different. After the subject pressed the key, the probe stimulus would immediately disappear and proceed to the next trial.

The experiment consisted of 3 groups, with 120 trials contained within each group. After the completion of each set of trials, the subjects were asked to rest for at least 30 s via a screen prompt to avoid fatigue effects.

### EEG recording

EEG data analysis was conducted using Matlab and letwave7. The preprocessing of EEG data involved applying a 30 Hz low-pass filter and re-referencing to the average of the left and right mastoid electrodes (M1 and M2). The time window of interest for EEG analysis spanned from 200 ms before the display of the memory array to the presentation of the probe stimulus, covering a window from −200 to 2,200 ms. Following the elimination of ocular artifacts via ICA component analysis, ±100 μV thresholds were set for artifact rejection at PO7/PO8 electrodes. Subsequent to aligning with the research objectives, additional analyses were performed utilizing the preprocessed waveforms. EEG data underwent separate averaging for distinct conditions, with a focus on the PO7/PO8 electrodes as the region of interest. To extract neural activity associated with the memorization-demanding squares, waveforms from the contralateral side were subtracted from the ipsilateral side. The resultant difference waves underwent correction for multiple comparisons using false discovery rate (FDR) correction (Benjamini and Hochberg, 1995) with a statistical threshold set at *p* < 0.05. Within the FDR-corrected time frames for statistical significance, fewer than five consecutive time sampling points were considered nonsignificant, while more than five consecutive time points were considered significant.

## Results

### Behavior results

**Figure 2:**
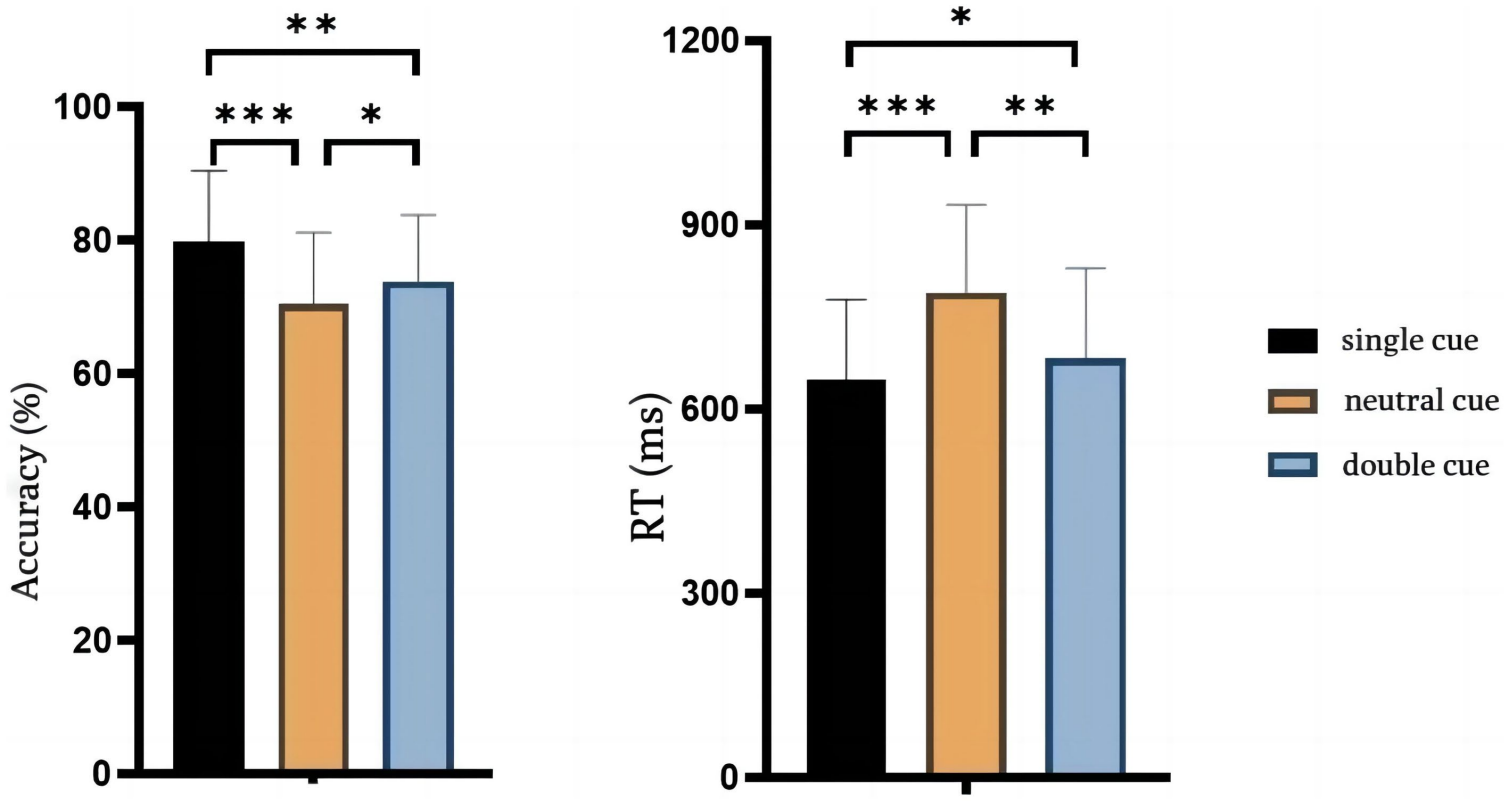
Behavior results of accuracy and RT. The left image is the result of accuracy in three condition and the right image is the RT result. Error bars reflect standard errors.

The correct rates and response times for the behavioral outcomes are shown in Figure 2. The paired-sample *t*-test showed that the correct rate of the single-cue was significantly higher than the neutral-cue [single-cue: 0.80 ± 0.03, neutral-cue: 0.70 ± 0.03; *t*(14) = 5.220, *p* < 0.001, Cohen’s *d* = 3.33] and the response time was significantly shorter than neutral-cue [single-cue: 649.4 ± 33.4, neutral-cue: 789.8 ± 37.1; *t*(14) = −6.264, *p* < 0.001, Cohen’s *d* = −3.97]. The correct rate of the double-cue was significantly higher than the neutral-cue [0.74 ± 0.03 for the double-cue; *t*(14) = 2.225, *p* < 0.05 = 0.043, Cohen’s *d* = 1.33], and the response time was significantly shorter than the neutral-cue [682.4 ± 38.1 for the double-cue; *t*(14) = −4.414, *p* < 0.05 = 0.001, Cohen’s *d* = −2.85]. The results indicated that the RCB effect was produced under both single and double-cue. In addition, the results also indicated that the accuracy of the single-cue was significantly higher than the double-cue [*t*(14) = 3.803, *p* < 0.05 = 0.002, Cohen’s *d* = 2] and had a significantly shorter response time than the double-cue [*t*(14) = −2.994, *p* < 0.05 = 0.033, Cohen’s *d* = −0.92]. Behavioral results indicated that RCBs generated under single-cue were significantly better than double-cue condition in terms of both accuracy [*t*(14) = 2.358, *p* < 0.05 = 0.0334] and response time [*t*(14) = 3.803, *p* = 0.002].

### EEG results

**Figure 3:**
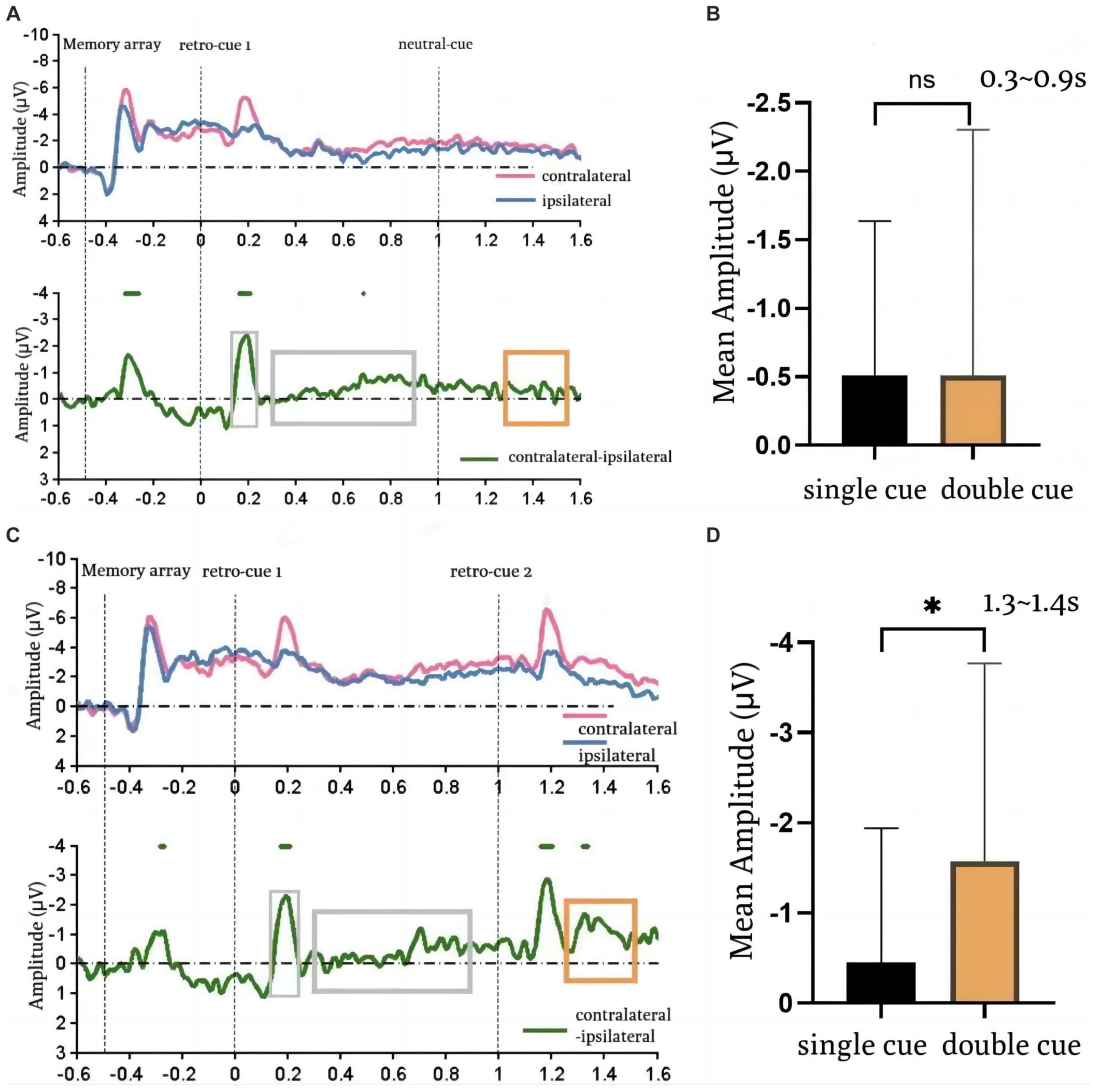
EEG result. **(A,C)** are the wave changes in the single-cue condition and the double-cue condition, respectively. **(B,D)** is the comparison of the single-cue and the double-cue on the first time window (0.3–0.9 s) and the second time window (1.3–1.4 s), respectively. Error bars reflect standard errors.

The EEG results are shown in Figure 3. The upper half shows: the average waveforms of the contralateral and ipsilateral sides at the PO7 and PO8 electrode sites for the single-cue and double-cue conditions in the mixed presentation. The contralateral and ipsilateral sides are relative to the field of view in which the color block array requiring memory was located. The lower half shows: the difference waveforms of the contralateral minus the ipsilateral side of the spatial cue in both conditions. From this, the CDA waveforms can be observed in the corresponding time periods. The delayed negative slow wave that appears between 0.4 ms and 0.8 ms after the appearance of the stimulus (retro-cue) is the CDA.

According to the relevant definitions of CDA, 0.3–0.9 s after the appearance of the first retro-cue (cue1-evoked CDA) and 1.3–1.4 s (cue2-evoked CDA) were selected as the time windows of interest, respectively, and the average amplitudes of the spatial cue difference waves were computed under the two conditions, and repeated-measurement ANOVAs were done between the two conditions for the two-time windows, respectively.

It was found that there was no significant difference in the mean amplitude of spatial cues in either condition during the first time window of interest, as shown in Figure 3. That is, there was no significant difference in the mean amplitude within the interval of CDA [single: −0.51 ± 0.29, double: −0.51 ± 0.46; *t*(14) = 0.003, *p* = 0.998, Cohen’s *d* = 0] induced after the presentation of the first cue regardless of whether it was a single-cue or a double-cue. This suggests that when participants have no idea if there was a second different retro-cue, after the first cue presentation, the cued item entered the active state and the other items were in the passive state. Within the second time window of interest (a comparison of mean amplitudes within the CDA window of interest is shown in Figure 3), a significant difference in mean amplitude was observed between the two conditions [single: −0.45 ± 0.38, dual: −1.58 ± 0.57; *t*(14) = 2.317, *p* = 0.036, Cohen’s *d* = 2.33]: the amplitude was significantly higher in the double-cue condition. This suggests that the CDA amplitude was higher in the double-cue condition compared to the single-cue condition, as new items entered the active state after the second cue prompt.

## Discussion

In the present study, we explored whether previously uncued items, when subsequently cued, produced an RCB effect, and the effects of different cue types on the storage status of items. Behavioral results showed that double-cue outperformed neutral-cues in terms of accuracy and response time, suggesting that double-cue also produced an RCB effect, which is consistent with previous findings (Rerko and Oberauer, 2013). In addition, the EEG results also showed a different storage status between single-cue condition and double-cue condition. There was a significant CDA wave amplitude in double-cue condition after the second cue compared to the single-cue condition, demonstrating that there was a movement of items from the passive state to the active state after the second cue. It is suggested that, if there was a 50% chance for the appearance of the second retro-cue and 100% probability its pointed item would be tested, the RCB effect occurs under double-cue because the remaining uncued items were not discarded or deleted when the first cue appeared, but were stored in the passive state. When the second cue appeared, it was able to transfer the cued item from the passive state to the active state, at which time the item was able to be further encoded and processed under internal attention. Thus, during detection, cued items were more advantageous under second internal attention than neutral-cues (where all items remain in working memory), and resulted in the RCB effect.

The results in present research is inconsistent with van Moorselaar’s et al. (2015) null effect, suggesting that cue distribution and the number of items in influencing the emergence of the retro-cue benefit (RCB) effect under double-cue conditions. As the proportion of double-cue diminishes, individuals tend to prioritize items cued by the initial cue, allocating a greater share of cognitive resources for processing, thereby leading to suboptimal resource allocation and subsequently yielding average extraction performance for uncued items. When the number of items to be processed surpasses working memory capacity, some items may fail to be effectively stored, mitigating the cue’s efficacy and potentially resulting in comparable performance between dual and neutral-cues. Moreover, the successful replication of Rerko and Oberauer’s (2013) findings underscores RCB as a robust effect within the retrospective detection paradigm. Notably, even when items have transitioned from a previously passive state (uncued) to an active state following subsequent internal attention, there is evidence to suggest that item encoding is enhanced, thereby improving accuracy and reducing response time during the detection phase.

The CDA results also supply some theoretical contributions to the “activity-silent” model. This research demonstrates that the item under the passive state can come back to the active state, which means that the status state in the VWM is sufficiently flexible and can change according to the internal attention. Specifically, if it is unclear whether there was a second retro-cue and pointed item that would be tested, individuals did not discard items unrelated to the ongoing task but rather shifted uncued items to a passive state. In this way, items can be processed and encoded when they are once again within the focus of attention, aligning with prior research. Previous studies have revealed that in the context of holding two items in working memory, only the item within the attentional focus consistently exhibited a bias toward visual attention and was behaviorally discernible, while the out-of-focus item failed to sufficiently bias attention for discernment. However, if the item was cued as relevant for a subsequent memory test, it was reintegrated into focus and exerted an attentional bias (Mallett and Lewis-Peacock, 2018). Similarly, research employing multivariate pattern analysis to decode brain activity observed that active representations of items in working memory reverted to baseline when attention shifted. Significant transcranial magnetic stimulation (TMS) reactivation effects and impacts on memory performance were evident only upon re-probing the item (Rose et al., 2016).

The current investigation echoes prior research, reiterating that the retro-cue benefit (RCB) effect under double-cue was less pronounced compared to single-cue, consistent with earlier findings (Landman et al., 2003; Matsukura et al., 2007; Rerko and Oberauer, 2013; van Moorselaar et al., 2015; Rhilinger et al., 2023). These observations suggest the efficacy of the first cue in directing internal attention. Notably, in Li and Saiki’s (2014) study, even when the second cue acted as a withdrawal cue, signaling the invalidity of the first cue, it exhibited superior performance compared to the neutral-cue when identifying the item indicated by the initial cue, thus retaining an RCB effect. Subsequent experiments within the same study further substantiated the enduring advantage of the first cue, demonstrating comparable outcomes between the first and second cues, outperforming the neutral-cue. Even when the first cue’s validity decreased to 17% and the second cue’s validity increased to 50%, the first cue continued to outperform the neutral-cue, consistently exhibiting a noteworthy RCB effect across all three experiments.

Our observation that single-cue yielded superior performance compared to double-cue further highlights the influence of the initial cue within the double-cue paradigm. In the context of our double-cue experiment, despite the complete invalidity of the first cue and subjects’ capacity to remove the item associated with the first cue from visual working memory upon the appearance of the second cue, results continued to indicate inferior performance under double-cue conditions in comparison to single-cue conditions—a finding suggesting the ongoing impact of the invalid first cue on overall performance. This aligns with recent evidence indicating a precision reduction for items shifted to a passive state relative to items consistently maintained in the active state (Rhilinger et al., 2023).

There are three possible reasons why the RCB effect for the double-cue was smaller than the single-cue. First, item representations declined over time, and attention was unable to fully improve these degraded representations when the second cue was present (Matsukura et al., 2007). Second, when multiple items were stored in VWM, it had been shown that those representations were not saved independently, but rather interacted with each other in a way that depends on attentional priority; specifically, high priority was not affected by low priority, but low priority received the influence of high priority items (Olivers, 2008). When the first cue was presented, the cued item had the highest priority, and the rest of the items were low priority; at this point, the items in the passive state were affected by the interference of the items in the active state, and when the items in the passive state re-entered the active state, their representation accuracy was reduced due to the interference. In addition, there was more than one storage item within the passive state, and there was some competition among them, making the item representations more fragile. Finally, after the appearance of the second cue, the second cue item then needed to be converted from the passive state to the active state. This transition imposed a certain conversion cost and consumed a certain amount of cognitive resources, and therefore, the second item was poorly represented under double-cue compared to single-cue.

The present study, while demonstrating that there is also an RCB effect for the second cue, still has some limitations. First, the present study argued that the different results seen in the van Moorselaar et al. (2015) and Rerko and Oberauer (2013) studies may be due to the proportion of different cue types and the excessive number of stores, the two variables were not controlled separately, and it was not clear whether it is specifically one of the variables or both variables together that influenced the second cue’s RCB effect. Future research could further explore the factors influencing the RCB effect of the second cue. Second, this study did not further explore the reason why the RCB was smaller in the double-cue than in the single-cue. Future research could further discuss whether this is due to the impairment of representation, item interference, or a shift in storage form. Finally, the conclusion that the second-cued item was preserved in the passive state instead of discarded may only established in our experiment settings. Future researchers could find the critical point that when second-cued items are discarded and when they are saved in the passive state.

Overall, the present study demonstrated that previously uncued items still produce an RCB effect when they are again under internal attention, suggesting some robustness of the effect. At the same time, this effect was again smaller under double-cue than single-cue, suggesting that there is some impact on the representation of items during shifts in their storage status.

## Data availability statement

The original contributions presented in the study are included in the article/supplementary material, further inquiries can be directed to the corresponding author.

## Ethics statement

Written informed consent was obtained from the individual(s) for the publication of any potentially identifiable images or data included in this article.

## Author contributions

RG: Writing – original draft. JW: Writing – review & editing. KF: Writing – original draft. QL: Writing – review & editing.
